# Supplementary material for: A high-resolution mRNA expression time course of embryonic development in zebrafish
Source: eLife. 2017 Nov 16;6:e30860. doi: 10.7554/eLife.30860 (PMC5690287; doi:10.7554/eLife.30860)
Supplement: Supplementary file 6. [file elife-30860-supp6.zip › biolayout-clusters-files/Cluster193-genes.html]

Cluster193


# Cluster193: Genes

| Ensembl ID | Gene Name | Chr | Start | End | Biotype |
| --- | --- | --- | --- | --- | --- |
| ENSDARG00000099376 | CTSZ | 2 | 54060512 | 54071869 | protein\_coding |
| ENSDARG00000045064 | ablim1b | 12 | 29931473 | 30105444 | protein\_coding |
| ENSDARG00000099003 | cyp2aa1 | 23 | 42468852 | 42486363 | protein\_coding |
| ENSDARG00000019418 | kcnj2a | 12 | 35586277 | 35587791 | protein\_coding |
| ENSDARG00000040568 | pdzd3a | 15 | 23515773 | 23532485 | protein\_coding |
| ENSDARG00000093954 | si:dkey-9i23.16 | 1 | 44239728 | 44244921 | protein\_coding |
| ENSDARG00000024560 | slc4a1b | 12 | 10016009 | 10062748 | protein\_coding |
